# Telerehabilitation After Anterior Cruciate Ligament Reconstruction Is Effective in Early Phases of the Recovery Programme

**DOI:** 10.3390/jcm14144843

**Published:** 2025-07-08

**Authors:** Bruno Turchetta, Giovanna Brancaleoni, Alessandro D’Alesio, Sara Tosoni, Marianna Citro, Matteo Turchetta, Lorenzo Polo, Ivan Pinna, Guglielmo Torre, Pier Paolo Mariani

**Affiliations:** 1 Top Physio Clinics, 00136 Rome, Italy; brunoturchetta@topphysio.it (B.T.); brancaleoni@toppphysio.it (G.B.); dalesio@topphysio.it (A.D.); tosoni@topphysio.it (S.T.); citro@topphysio.it (M.C.); matteoturchetta1@gmail.com (M.T.); 2 Villa Stuart Sport Clinic—FIFA Medical Centre of Excellence, 00136 Rome, Italy; polo@brain-srl.com (L.P.); ivanpinna@live.it (I.P.); ppmariani@virgilio.it (P.P.M.); 3 Department of Movement, Human and Health Sciences, University of Rome “Foro Italico”, 00136 Rome, Italy

**Keywords:** ACL, telerehabilitation, knee surgery, sport

## Abstract

**Background/Objectives**: In recent years, scientific literature has illustrated the growing interest in telerehabilitation after ACL reconstruction. The aim of this study is to compare the effectiveness of remotely supervised rehabilitation with traditional supervised rehabilitation after ACLR, focusing on objective postoperative functional assessment outcomes. **Methods**: A retrospective analysis of prospectively collected data was carried out, selecting patients that underwent arthroscopic ACLR by a single surgeon. Functional assessments of the patients were carried out at 1 and 2 weeks and 1, 2 and 3 months after surgery, including range of motion (ROM), maximal voluntary isometric contractions (MVICs) of extensor and flexor muscles, the sit-to-stand test and the countermovement jump. Intergroup statistics were carried out using a non-inferiority hypothesis. **Results**: A total of 251 patients were included in this study (supervised rehabilitation *n* = 165; remotely supervised rehabilitation *n* = 86). Functional assessment improved over time in both groups. The extension ROM deficit decreased to 0 difference 30 days after surgery. The median flexion ROM ILD at 60 days was significantly different among the groups, with a residual 10° ILD in the Group R compared with 0° ILD in group S (*p* = 0.01). All other assessments did not achieve statistical significance. **Conclusions**: The results support the integration of a digital rehabilitation tool in post-ACLR recovery programs. The results suggest that remotely supervised rehabilitation can be a viable alternative to traditional supervised rehabilitation for early-stage recovery. However, more research is needed to optimize protocols and to identify patients who may benefit most from this approach.

## 1. Introduction

Anterior cruciate ligament (ACL) injury is the most common among sport injuries, with a rupture rate ranging between 0.005 and 0.2 ruptures for each 1000 h of sport practice [1], as diagnosed by clinical assessment and MRI evaluation [2]. Although conservative treatment may be advocated for certain patients with low functional requests, the mainstay of treatment is surgical reconstruction of the ACL using an autologous tendon graft [3]. This procedure is demanding for the whole joint and the surrounding tissues as it involves the harvesting of the patellar tendon or hamstring graft, leading to a muscular deficit and imbalance that will need to be addressed over the postoperative period. The main objectives of early-stage rehabilitation after an ACL reconstruction are (i) full range of motion (ROM) restoration, (ii) resolution of joint effusion and (iii) restoration of interlimb strength symmetry. These goals must be met in the shortest possible time to avoid postoperative complications and provide the quickest return to full daily activities and sport participation [4,5,6].

In the first 15 days postoperatively, most of the patients experience a reduction in their range of motion (ROM) as well as knee swelling and difficulties in accurately following the rehabilitation program. For this reason, the supervision of a physiotherapist is generally advised during the whole rehabilitation pathway [7,8].

In the last decade, attention to telerehabilitation has increased, particularly after the COVID-19 pandemic; the importance of time-effectiveness and cost-efficiency were taken into higher consideration. According to the current literature, telerehabilitation is used to treat several clinical conditions among neural, musculoskeletal and cardiopulmonary disorders [9,10]. Furthermore, some evidence supports home-based telerehabilitation (HBTR) as a valid alternative to center-based rehabilitation (CBR), reporting comparable or superior outcomes especially in terms of patient compliance, functional rehabilitation and cost-efficiency [11]. The main concern about HBRT is not only the lack of real-time supervision by a professional physiotherapist but also the unavailability of advanced strengthening exercises using gym machines that could compromise appropriate strength recovery. However, the home-based unsupervised rehabilitation definitely differs from the home-based remotely supervised program, where a physiotherapist can interact in semi-real time with the patient through a remote monitoring system. In addition to this, the development of appropriate strength recovery protocols with home accessories may supplement a gym-based strengthening program for efficient strength outcomes.

Some telerehabilitation software suites have recently been developed that support the home-based, remotely supervised approach and have been shown to positively impact the home-based rehabilitation programs regarding total hip and knee arthroplasty [12,13].

In 2020, Busso et al. [12] designed a randomized, controlled parallel-group open-label trail with blinded assessors of 56 patients who had undergone primary total hip arthroplasty (THA). They compared a standard 2 weeks in clinic rehabilitation followed by 3 weeks of unsupervised home-based rehabilitation to a standard 2 weeks in clinic rehabilitation followed by 3 weeks of home-based ReHub-assisted telerehabilitation. Notwithstanding the short intervention duration and the lack of long-term follow-up, they found that the ReHub-assisted telerehabilitation group performed better than the standard rehabilitation group in the majority of physical performance assessments (Time-up and Go Test, Hip Range of Motion, Hip Muscle Strength Assessment) and obtained higher scores in terms of the Independence level scale (Functional Independence Measure Scale), pain intensity (Numerical Rating Scale), hip disability (Hip Disability and Osteoarthritis Outcome Score) and the patients’ perceptions of clinical improvement (Global Rating of Change Scale).

A recent study from Nuevo et al. [13] randomized 52 patients who received total knee arthroplasty (TKA). They were divided into two groups; the control group received standard rehabilitation upon discharge, which consisted of a 4-week plan of five daily exercises and up to 10 physiotherapy home visits, while the ReHub-user group performed the daily exercises autonomously. They compared the results in terms of range of motion (ROM), walking ability (Time-Up and Go Test, maximal isometric voluntary contraction of the quadriceps of the operated leg (MIVC) and the maximal isometric voluntary contraction of the hamstring of the same leg. They found no statistically significant difference in most of the outcomes apart from the isometric strength of the quadriceps and higher adherence to the rehabilitation protocol.

Despite these several promising results, there is a lack of specific studies directly comparing telerehabilitation using a web-based application with traditional supervised rehabilitation in post-ACL reconstruction patients measuring functional factors (range of motion recovery, effusion resolution, strength recovery) [14,15,16]. In particular, the present study is the first one to assess the results of home-based, remotely supervised rehabilitation in ACLR patients, according to our best knowledge, and is the first to focus on postoperative functional measures, including ROM and strength recovery.

The aim of this comparative, non-randomized study is twofold: first, to compare the effectiveness of remotely supervised rehabilitation using a web-based application with traditional assisted rehabilitation in patients undergoing ACL reconstruction and second, to highlight the differences between the two treatment approaches both in the short- and the mid-term. We hypothesized that functional outcomes in the very short- and short-term (up to 1 month postoperatively) would be similar between the groups, while the difference between the two groups would rise in the mid- and long-term (1 month after surgery).

## 2. Materials and Methods

### 2.1. Patients and Design

**Figure 1:**
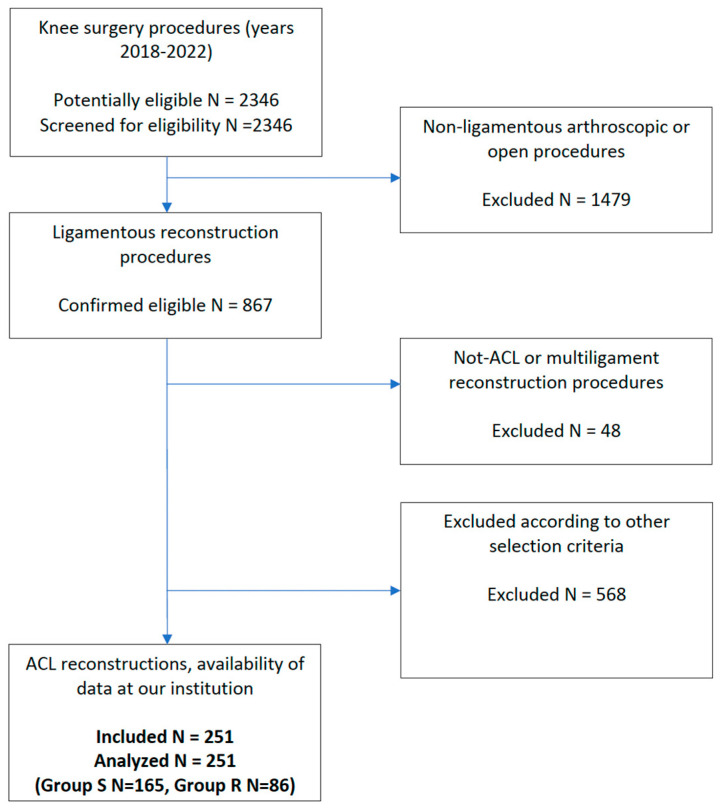
Flow diagram of the population selection process.

A retrospective analysis of prospectively collected data was carried out, selecting patients that underwent arthroscopic ACL reconstruction by a single surgeon (PPM) from January 2018 to December 2022 at the Villa Stuart Sport Clinic, FIFA Medical Centre of Excellence. From the years 2018–2022, the senior author performed a total of 2346 arthroscopic knee procedures. Of these, 867 were ligamentous procedures, including 766 ACL reconstruction procedures (Figure 1). The patients, according to their preferences and residence locations were followed up with for rehabilitation programs, either in supervised sessions at our physiotherapy facilities (Top Physio Clinics) or by using a remote-based telerehabilitation software (ReHub DyCare, Bio-sensing Solutions SL, Barcelona, Spain). Data extracted from records included demographic data of the cohort, activity level (Tegner scale), surgical details (type of graft), patient-reported outcomes and postoperative functional evaluations. Exclusion criteria were any previous index knee surgery, absence of relevant orthopedic pathology that prevented the execution of the rehabilitation program, associated meniscal procedures that required restricted weight-bearing in the first weeks or month after surgery, patient lost at clinical follow-up before 3 months postoperatively, and daily adherence lower than 85% to the remote rehabilitation exercise program.

### 2.2. Functional Evaluation Protocol

Functional assessments of patients were carried out at all follow-up steps (1 and 2 weeks and 1, 2 and 3 months after surgery) by an assessor who was blinded to the rehabilitation protocol followed by the patient (and therefore blinded to group allocation). At 1 and 2 weeks after surgery, knee girth, flexion-active range of motion (aROM) and extension ROM were measured and compared with contralateral (absolute difference in cm and in degrees, respectively). At 1 month and 2 months, the maximal voluntary isometric contractions (MVICs) of the extensor and flexor muscles were measured in addition to the previous measurements. The assessed MVICs were compared with the contralateral (expressed in N), and the Limb Symmetry Index (LSI) was calculated by dividing the absolute value obtained from the operated limb by the absolute value obtained from the healthy limb, and the result was multiplied by 100 (interlimb difference expressed as %). At the last follow-up, the sit-to-stand test and the countermovement jump were assessed in addition to the previous evaluations. For these evaluations, the LSI was also calculated, and the interlimb difference was expressed in %. For all patients, the International Knee Documentation Committee (IKDC) scores were collected at the baselines and last follow-ups.

### 2.3. Surgical Technique

The ACL reconstruction (ACLR) was carried out in all patients by the same sport trauma surgeon (P.P.M.), who has over 40 years of experience in knee arthroscopy and ligament reconstruction. The ACL was reconstructed with the trans-tibial technique, using either a bone–patellar tendon–bone (BPTB) or a quadrupled hamstring (HS) graft. Associated meniscal surgery may vary depending on intraoperative findings and included selective meniscectomy; all-inside Morgan sutures for ramp lesions; and all-inside sutures were used for peripheral longitudinal tears. One intraarticular drainage each was placed for those patients who had ACLR with HS, and one additional suprafascial drainage each was placed for those receiving surgery with BPTB.

### 2.4. Rehabilitation Protocol (Non-Group Dependent)

Full weight bearing was allowed on the first postoperative day after the removal of drainages, with walking and stair climbing allowed with the use of an extension-locked knee brace (Rehabrace, FGP, Dossobuono (VR), Italy). A Continuous Passive Motion (CPM) machine was prescribed with 4 to 6 sessions per day in the first two postoperative weeks, with a passive ROM (pROM) recovery within 0–90° in the first week and 0–110 in the second week.

### 2.5. Rehabilitation Protocol (Supervised Group)

Unloaded isometric exercises for quadricep and stretching of flexor muscles were initiated from the second postoperative day. From the third week, active flexion and extension were encouraged for the full recovery of aROM including using a progressive-resistance bike and squat exercises within 90° of flexion. From the second postoperative month, progressive-resistance isometric and isotonic strength exercises for extensor muscles were allowed. Running on a treadmill was allowed from the third month onward if the walking scheme was complete and unbiased. Progressive agility exercises were allowed within the third month.

### 2.6. Rehabilitation Protocol (Remote Group)

Milestones were similar to those for Group S. In Group R, exercises were initiated on the second postoperative day after discharge from the clinic, using the ReHub platform (Bio-sensing Solutions SL, Barcelona, Spain), a telerehabilitation digital system specifically designed for musculoskeletal rehabilitation that allows the patient to be monitored remotely by a physiotherapist. Each patient was provided with an inertial motion sensor unit (IMU) that was integrated with the web-based application. The sensor was a lightweight, wireless device that transmits kinematic data in real time to the platform via Bluetooth, enabling both patient feedback and remote monitoring.

The sensor placement was standardized: for the quadriceps isometric voluntary-contraction exercises, the sensor was positioned 10 cm proximal to the patella (middle thigh, corresponding to the rectus femoris). No sensor was needed for the isometric contraction of the glutes or for the mobilization of the patella.

Each exercise (see Table 1) was assigned through the platform with a video demonstration and detailed parameters, and biofeedback was displayed in real time thanks to data shared by the IMU. All data (number of sets, repetition times, adherence rates, and quality of execution) were automatically recorded and stored on a cloud server, allowing remote access for physiotherapists and clinical supervisors. Adherence and progression were evaluated weekly via video call. Adjustments to the protocol were made based on real-time sensor data and clinical judgment. The patients had also the option to contact the physiotherapist through a chat system embedded in the software.

### 2.7. Data Management and Statistical Analysis

Data were collected into a database, with an edit check and validation before data analysis. STATA software version 18 (StataCorp, College Station, TX, USA) was used for all computational analysis. A baseline intergroup heterogeneity assessment was carried out. Continuous variables were reported as mean and standard deviation (SD), discrete variables as median and interquartile range (IQR) and categorical variables as raw frequency and percentage. Student’s *t* test for unpaired data and the Wilcoxon-Mann–Whitney test were used for mean comparisons of interlimb differences (ILD, either absolute or percentage) between the groups, using a non-inferiority hypothesis. The Kruskal–Wallis test or the Analysis of Variance (ANOVA) with the Bonferroni correction were used to assess the improvement of functional evaluation parameters at follow-up. A significance threshold was set with *p* = 0.05 (95% CI). A post hoc power estimation was carried out using the intergroup difference in MVICs at one month after surgery as a main endpoint to calculate the effect sizes (Cohen’s D = 0.45). A power value of 93% was obtained for the number of patients included.

### 2.8. Ethical Considerations

Investigators collected only data regarding common clinical practice, which were used for diagnosis and full monitoring during and after the surgical treatment. The Institutional Review Board approved the observational study. All patients signed informed consent for data collection, to be used for research purposes. The investigation was carried out under the guidelines of the Declaration of Helsinki and according to the standards of good clinical practice.

## 3. Results

### 3.1. Cohort Demographics

A total of 251 patients was included in this study. Of these, 165 carried out a supervised rehabilitation protocol (Group S), and 86 carried out a remotely supervised protocol (Group R). The patients included had a mean age of 29.7 ± 13 years. The median Tegner activity scale was 6 (IQR 3, 7). The patients underwent ACLR with BPTB in 57 cases and with HS in 106 cases. The IKDC score at baseline averaged 46 ± 13.7 in Group R and 56.7 ± 18.4 in Group S at baseline. All these parameters were comparable between groups at a preliminary intergroup heterogeneity test (See Table 2).

### 3.2. Functional Assessment Outcomes

**Figure 2:**
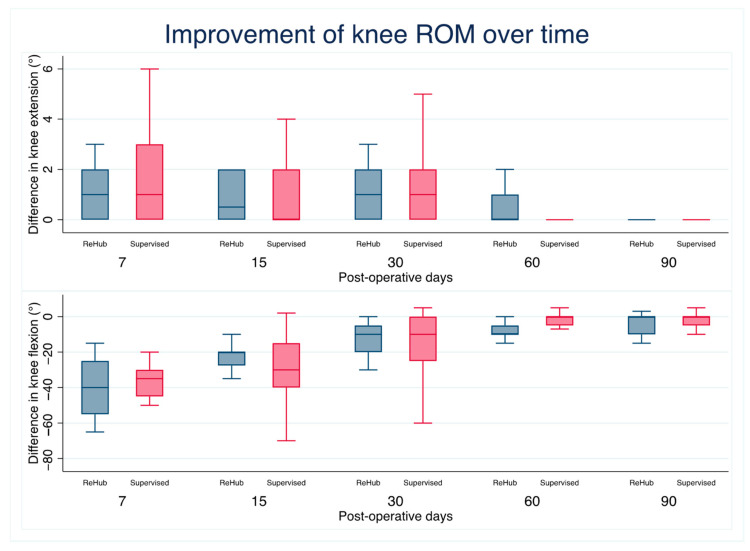
aROM improvement over time: both groups reached the same difference in knee flexion and extension at 90-day follow-up.

**Figure 3:**
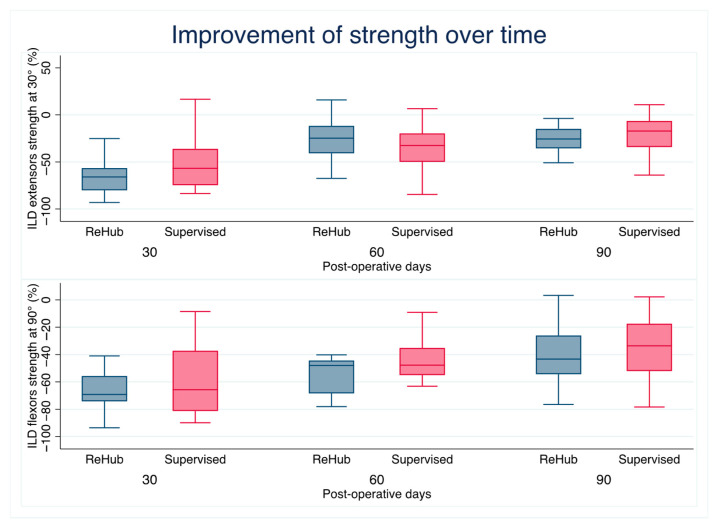
MVIC improvement over time: both groups reached the same difference in extensor and flexor strength at 90-day follow-up.

All functional parameters improved over time in both groups. The extension aROM deficit decreased to 0° ILD (complete interlimb symmetry) 30 days after surgery on average in both groups. The median flexion aROM ILD at 60 days was significantly different between groups, with a residual 10° ILD in Group R compared with 0° ILD in Group S (*p* = 0.01); see Figure 2. At the last follow-up, the IKDC score averaged 63.7 ± 16 in Group R and 69.3 ± 12 in Group S, and the difference was not statistically significant (*p* = 0.13). All other functional assessment measures did not achieve statistical significance; see Table 3 and Table 4 and Figure 3.

## 4. Discussion

The main findings of the current investigation indicate that traditional supervised center-based rehabilitation and remotely supervised home-based rehabilitation approaches led to significant and comparable improvements in functional outcomes over time, aligning with the primary objectives of early-stage rehabilitation, namely, the restoration of aROM, resolution of joint effusion and recovery of interlimb strength symmetry both in short- and medium-term follow-ups. The comparable improvements observed in several key parameters, such as the resolution of extension aROM deficit by 30 days post-surgery, suggest that remotely supervised rehabilitation can be a feasible alternative to traditional supervised rehabilitation, particularly in the early phases of recovery. This observation is consistent with a growing body of literature supporting the use of telerehabilitation for various musculoskeletal conditions, highlighting its potential to enhance patients’ access to care and to improve cost-efficiency [12,13].

However, the statistically significant difference observed in the median flexion ROM interlimb difference (ILD) at 60 days, with the remote-rehabilitation group exhibiting a residual 10° ILD compared with the supervised group, warrants careful consideration. Several factors could potentially explain the observed difference in flexion ROM recovery. Traditional supervised in-clinic rehabilitation often involves manual therapy techniques, such as joint mobilization and myofascial release, which can directly address joint stiffness and muscle tightness, thereby facilitating aROM improvement [17]. Furthermore, the close supervision and guidance provided by a physiotherapist in a clinical setting allow for real-time adjustments to the rehabilitation program based on the patient’s individual progress and response. This personalized approach may be more challenging to replicate fully in a telerehabilitation setting, in which interventions are typically delivered remotely through video consultations and exercise demonstrations.

A systematic review by Papalia et al. [18] collected studies about home-based unsupervised rehabilitation after ACLR, showing that both the home-based or the supervised rehabilitation approach are effective for functional recovery up to long-term follow-up. However, all the included studies reported clinical outcomes in terms of patient-reported measures (mainly IKDC and Lysholm scores). Indeed, the present study is the first one to evaluate the effects of remotely supervised rehab on postoperative objective functional assessment in terms of ROM and strength recovery.

High-quality studies about home-based rehabilitation platforms (with remote supervision) are lacking, especially in post-ACLR rehabilitation. Previous studies on total hip and knee arthroplasty [12,13] have demonstrated that telerehabilitation can offer comparable clinical outcomes to traditional in-person care, especially in terms of patient compliance and cost-efficiency. Our findings align with these conclusions while also highlighting a potential limitation regarding the recovery of flexion ROM in ACLR patients undergoing remote rehabilitation. Recent findings from Lim et al. [19] showed that patients who underwent ACLR and received home-based augmented reality rehabilitation had similar results compared to those who received brochure-based rehabilitation. Another study from Liao et al. [20], carried out during the COVID-19 pandemic, found that home-based telerehabilitation yielded results comparable to face-to-face rehabilitation in terms of functional outcomes and patient-reported outcome scores. Another recent study protocol from Alegrete et al. [21] reported that patients who received home-based telerehabilitation are expected to recover better than those who received standard in-clinic rehabilitation at the end of the trial. Several strengths support the validity of this investigation, including a relatively large cohort, consistency in surgical technique performed by a single experienced operator and serial functional assessments based on objective, reproducible measures. The findings of the present study should be interpreted in the context of some limitations. The retrospective nature of the analysis and the non-randomized allocation of patients to the two rehabilitation groups introduce the potential for selection bias and confounding variables. While the authors report comparable baseline characteristics between the groups, it is possible that other unmeasured factors, such as several patient-reported outcome measures (PROMs)—patient motivation, adherence to the rehabilitation program or preexisting psychological factors—may have influenced the outcomes. A randomized controlled trial (RCT) design would provide stronger evidence for the comparative effectiveness of telerehabilitation by minimizing the risk of bias and ensuring a more balanced distribution of patient characteristics across the study groups in order to better stratify and control for those confounding factors that may relevantly impact the results.

Furthermore, this study’s focus on functional outcomes, while important, does not fully capture the patient experience and the broader impact of telerehabilitation. Future research should consider incorporating patient-reported outcome measures (PROMs), such as pain levels, functional disability scores and satisfaction with the rehabilitation process, to provide a more comprehensive evaluation of telerehabilitation’s effectiveness. Additionally, long-term follow-up studies are needed to assess the sustainability of the observed benefits and to determine whether any differences between the two rehabilitation approaches emerge over time, particularly in terms of return to sport and the incidence of re-injury. Indeed, in the medium- and long-term follow-up after ACL surgery, different recovery features became observable, with a focus on proprioception, agility and explosive muscular power. However, the recovery of these features is mainly driven by on-field exercises rather than by gym rehabilitation; therefore, remote training for these muscular properties would need further investigation.

## 5. Conclusions

In conclusion, this study supports the integration of a digital rehabilitation tool with a remotely supervised approach in post-ACLR recovery programs. Taking into account the limitations of this study, the results may suggest that telerehabilitation could be a viable alternative to traditional supervised rehabilitation. This result is in accordance with the existing literature, although this study focused specifically on objective functional measurements that have never been investigated before. The observed difference in flexion ROM recovery highlights the need for further research to optimize protocols and identify patients who may benefit most from this approach, possibly defining some key intermediate assessment tools that may help to prevent functional imbalances, including ROM deficit. Future studies should focus on addressing the limitations of the current study design, exploring the most appropriate protocols in telerehabilitation and investigating the long-term impacts of telerehabilitation on patient-reported outcomes and patients’ returns to sports.

## Figures and Tables

**Table 1:** Exercise summary.

Exercise	Target Area	Repetitions	Sets	Time Per Rep (s)	Rest Between Sets (s)
Patellar mobilization—left knee	Patellofemoral joint	1	3	60	0
Gluteus isometric contraction (supine)	Gluteal muscles	10	5	9	30
Plantar flexion with elastic band—left ankle	Calf muscles, ankle ROM	10	5	5	20
Quadriceps isometric contraction—left knee	Quadriceps femoris	10	5	9	30
Straight-leg isometric hold—left leg	Quadriceps + hip flexors	10	10	9	30

**Table 2:** Demographic details.

Variable (Synthesis Measure)	Total	Group R	Group S	*p* Value ^1^
N.er	251	86	165	-
Sex (M/F)	106/57	58/27	48/30	0.37
Age (mean ± SD)	29.7 ± 13	30.9 ± 13.2	28.4 ± 12.8	0.466
Tegner (median (IQR))	6 (3, 7)	6 (3, 7)	7 (5, 7)	0.102
IKDC (mean ± SD)	53.1 ± 17.5	46 ± 13.7	56.7 ± 18.4	0.089
Type of graft (BPTB/HS)	57/106	26/59	31/47	0.221

(^1^) Intergroup comparison for heterogeneity (Chi^2^ test, Student’s *t* test).

**Table 3:** Intergroup comparison for functional evaluation (flexion and extension aROM and knee girth) at follow-up.

	Postoperative Days					
	7	15	30	60	90	*p* Value ^1^
**Extension aROM ILD (°)**						
Supervised						
Median	1	0	1	-	-	0.003 *
First quartile	0	0	0	-	-	
Third quartile	3	2	2	-	-	
Remote						
Median	1	0.5	1	-	-	0.13
First quartile	0	0	0	-	-	
Third quartile	2	2	2	-	-	
Total						
Median	1	0	1	-	-	0.003 *
First quartile	0	0	0	-	-	
Third quartile	3	2	2	-	-	
*p* value intergroup ^2^	0.86	0.78	0.84			
**Flexion aROM ILD (°)**						
Supervised						
Median	−35	−30	−10	0	0	0.001 *
First quartile	−45	−40	−25	−5	−5	
Third quartile	−30	−15	0	0	0	
Remote						
Median	−40	−20	−10	−10	0	0.001 *
First quartile	−55	−27.5	−20	−10	−10	
Third quartile	−25	−20	−5	−5	0	
Total						
Median	−39	−25	−10	0	0	0.001 *
First quartile	−45	−35	−20	−10	−5	
Third quartile	−30	−15	−5	0	0	
*p* value intergroup ^2^	0.41	0.39	0.98	0.01 *	0.37	
**Knee girth ILD (cm)**						
Supervised						
Median	3.5	3	1.75	-	-	0.13
First quartile	0.5	2	1	-	-	
Third quartile	4	3.5	3	-	-	
Remote						
Median	3	3	2	-	-	0.18
First quartile	2.5	2	1	-	-	
Third quartile	5	3.75	3.5	-	-	
Total						
Median	3	3	1.75	-	-	0.014 *
First quartile	2	2	1	-	-	
Third quartile	5	3.5	3.25	-	-	
*p* value intergroup ^2^	0.44	0.59	0.77			

^1^ Kruskal–Wallis one-way nonparametric ANOVA. ^2^ Wilcoxon–Mann–Whitney U test. (*) Significant.

**Table 4:** Intergroup comparison for functional evaluation (interlimb difference (ILD) for strength, sit-to-stand test and countermovement jump peak torque) at follow-up.

	Postoperative Days					
	7	15	30	60	90	*p* Value ^1^
**Extension MVIC at 30° ILD (%)**						
Supervised						
Mean	-	-	−50.25	−35.55	−21.65161	0.009 *
Standard deviation	-	-	33.17275	23.13612	23.85171	
Remote						
Mean	-	-	−63.65454	−26.33077	−29.34706	0.0009 **
Standard deviation	-	-	25.01569	27.23406	22.23376	
Total						
Mean	-	-	−58.01053	−32.47692	−24.37708	<0.001 **
Standard deviation	-	-	28.66771	24.61223	23.35059	
*p* value intergroup ^2^			0.32	0.28	0.28	
**Flexors MVIC at 30° ILD (%)**						
Supervised						
Mean	-	-	−58.3375	−44.968	−33.46333	0.014 *
Standard deviation	-	-	29.09187	21.7821	20.8359	
Remote						
Mean	-	-	−64.51818	−51.10769	−40.78235	0.019 *
Standard deviation	-	-	19.35607	20.81195	21.56647	
Total						
Mean	-	-	−61.91579	−47.06842	−36.11064	0.0001 **
Standard deviation	-	-	23.39025	21.37635	21.16857	
*p* value intergroup ^2^			0.58	0.41	0.26	
**Sit-to-stand test ILD (%)**						
Supervised						
Mean	-	-	−35.225	−23.852	−16.16	0.0009 **
Standard deviation	-	-	15.50569	15.62792	13.24601	
Remote						
Mean	-	-	−31.56667	−26.26154	−19.7625	0.19
Standard deviation	-	-	12.40504	13.6395	11.9653	
Total						
Mean	-	-	−34.22727	−24.67632	−17.41304	0.0001 **
Standard deviation	-	-	14.62926	14.83572	12.6604	
*p* value intergroup ^2^			0.53	0.64	0.52	
**Countermovement jump peak torque ILD (%)**						
Supervised						
Mean	-	-	-	-	−18.04706	-
Standard deviation	-	-	-	-	15.84784	-
Remote						
Mean	-	-	-	-	−8.725	-
Standard deviation	-	-	-	-	17.44465	-
Total						
Mean	-	-	-	-	−16.27143	-
Standard deviation	-	-	-	-	16.14435	-
*p* value intergroup ^2^					0.31	

^1^ One-way ANOVA (Bonferroni comparison). ^2^ Unpaired Student’s *t* test, non-inferiority (Pr. |T| > |t|). (*) Significant. (**) Highly significant.

## Data Availability

Data are available upon a motivated request by the corresponding author.

## Abbreviations

The following abbreviations are used in this manuscript:

ACL	Anterior Cruciate Ligament
ACLR	Anterior Cruciate Ligament Reconstruction
aROM	Active Range of Motion
BPTB	Bone-Patellar Tendon-Bone
CBR	Center-Based Rehabilitation
CPM	Continue Passive Motion
HBTR	Home-Based Telerehabilitation
HS	Quadrupled Hamstring
IKDC	International Knee Documentation Committee
ILD	Interlimb Differences
IQR	Interquartile Range
LSI	Limb Symmetry Index
MVIC	Maximal Voluntary Isometric Contraction
pROM	Passive Range of Motion
PROMs	Patient-Reported Outcome Measures
RCT	Randomized Controlled Trial
ROM	Range of Motion
SD	Standard Deviation
THA	Total Hip Arthroplasty
TKA	Total Knee Arthroplasty
